# Using EMDR therapy with patients in an acute mental health crisis

**DOI:** 10.1186/s12888-019-2426-7

**Published:** 2020-01-09

**Authors:** Simon Proudlock, Jasmine Peris

**Affiliations:** 1 Prospect Park Hospital, Honey End Lane, Tilehurst, Reading, RG30 4EL UK; 2 School of Psychology, University of Surrey, Guildford, Surrey, GU2 7XH UK

**Keywords:** Suicide, Mental health crisis, EMDR therapy, Inpatient, CRHTT, Trauma

## Abstract

**Background:**

Death by suicide continues to be a global public health concern with little research demonstrating the effectiveness of treatment options. This exploratory study exams the efficacy of Eye Movement Desensitisation and Reprocessing (EMDR) Therapy delivered to patients experiencing an acute mental health crisis to explore if by treating their background trauma, improvements could be seen in their general psychopathology and if there was a resulting decrease in their desire for suicide.

**Methods:**

A practice-based service development project was conducted within a mental health hospital. A non-randomised, exploratory pre-test post-test design was utilised. Participants were identified from adult patients currently receiving care from either an inpatient mental health ward or the Crisis Resolution and Home Treatment Team (CRHTT). Those who had reported experiencing at least one event that they considered to be traumatic were offered EMDR Therapy. Notes from the electronic database were analysed to assess contact with services 12 months prior to treatment and following treatment.

**Results:**

72 patients were offered treatment in the study with 57 completing treatment. Patients made significant improvements across all the psychometrics, including a reduction in suicidal ideation. The majority needed less than 10 sessions and needed no onward referral for further psychological therapy. Cost savings were realised by retracting referrals for further therapy and in early discharge from CRHTT and acute wards, and by preventing admissions. Contact with services post treatment also reduced.

**Conclusions:**

EMDR Therapy can be an effective treatment for patients experiencing a mental health crisis who have a trauma picture, resulting in significant improvements in their mental well-being and substantial cost savings for the National Health Service (NHS).

## Background

Death by suicide continues to be a leading cause of premature death across the globe. More than 55,000 suicides occur in the European Union each year, including on average around 6000 in the United Kingdom (UK). Suicide is the leading cause of death among young people aged 20–34 with men aged 45–49 at highest risk of completed suicide. Suicide rates are continuing to rise in the under 25 age group [1]. A similar situation is occurring in the United States of America which has seen a substantial increase in the suicide rates over the preceding 15 years [2].

Suicides in patients under the care of mental health services have also increased in recent years with as many as 28% of those who complete suicide concurrently receiving treatment from mental health services [3]. The loss of a person by suicide has a significant emotional impact on friends and families, as well as an estimated economic and social cost in the region of £1·7million per death [4].

In the UK, people at risk of suicide and other acute mental health difficulties are treated under the care of inpatient mental health hospitals or Crisis Resolution and Home Treatment Teams (CRHTT) [5]. These services are intended as limited periods of care and many patients are discharged to waiting lists for further psychological treatment. Psychological therapy is rarely available within these services. As waiting times increase, so does a patient’s propensity for repeated crises and continued destabilisation. This can result in a catastrophic effect on an individual’s employment, relationships, housing and general health and has a large economic impact on NHS acute services and the cost of ongoing psychological treatment.

Suicide prevention is high on the national agenda [6] and timely intervention for an individual experiencing a mental health crisis is imperative to help prevent death by suicide. The National Institute of Health and Care Excellence (NICE) provides no formal guidance on treating suicidality but gives comprehensive guidance aimed at preventing suicide [7]. Despite this, there is widespread evidence for the effectiveness of Cognitive Behavioural Therapy (CBT) with individuals who are suicidal [8–12] and Dialectical Behavioural Therapy (DBT) [13–15]. Eye Movement Desensitisation and Reprocessing (EMDR) itself occurred twice in the literature [16, 17] with Solution Focused Therapy [18–20] reporting some encouraging results both individually and within groups.

A number of meta-analyses and systematic reviews have supported these findings and concluded that adults who received psychological therapies such as CBT, DBT and problem-solving therapy decrease the repetition of their self-harm and suicide attempts [21–23]. These findings are consistent with research from inpatient mental health settings [24–26]. Within an emergency department setting (e.g. Accident and Emergency), it was reported that ‘active contact’ and follow up interventions were successful in preventing a repeat suicide attempt [27].

A significant body of evidence demonstrates that an experience of psychological trauma increases the risk for suicide [28–31]. Despite this, only a few studies have examined the treatment of Post-Traumatic Stress Disorder (PTSD) and a comorbid presentation of suicidal ideation [32, 33]. A review of the literature on the association of PTSD and past and current suicidal ideation found that PTSD was associated with an increased risk of suicidal ideation and prior attempts, but found little to suggest this led to completed suicides. The review also found that PTSD is rarely mentioned in the literature on suicide, and vice versa; risk of suicide is rarely mentioned in the literature related to trauma [34]. More recent research has indicated a strong link between childhood trauma and an increased risk of suicide [35, 36]. Historically such patients have been routinely excluded from PTSD studies as they are perceived as too risky [37], adding to the myth that suicidal patients are not safe to be treated [38].

EMDR Therapy is an empirically validated intervention for trauma and uses a comprehensive eight-phase protocol. It is guided by the adaptive information processing (AIP) model in which present symptoms are seen as unprocessed memories stored in the brain that lead to maladaptive information processing and present as symptoms of posttraumatic stress disorder [39]. EMDR Therapy asserts that following treatment of a traumatic memory, information processing is enhanced and new associations are forged, resulting in new learning, elimination of emotional distress, and development of cognitive insights.

EMDR Therapy has been found to be an efficacious treatment for PTSD [39–41] as well as for processing other emotional memories [42]. More recently its clinical applications have grown to include anxiety, depression, complex trauma, health related problems, body dysmorphic disorder, substance misuse, bipolar disorder, chronic back pain and PTSD underlying psychosis [43, 44].

However, little research has been conducted into the efficacy of EMDR with patients in acute mental health services at high risk of suicide. One paper described a retrospective case series of a number of patients who were treated using EMDR within a CRHTT, with the researchers finding that taking a trauma informed approach reduced the risk of suicide and prevented further presentations to a CRHTT [45]. More recently, EMDR has found to reduce the severity of suicidal thoughts in patients with major depressive disorder treated in an inpatient setting [16].

The aim of this study was to use a practice-based service development project to examine if EMDR Therapy is effective in an acute inpatient ward or CRHTT. It is hypothesised that on completion of treatment participants will experience less anxiety, depression and symptoms of PTSD and experience a decrease in their desire for suicide, as well as be better placed to manage their mental health.

## Methods

### Study design and participants

A practice-based service development project was conducted within a mental health hospital which involved offering an established therapy (EMDR Therapy) in a new setting. A non-randomised, exploratory pre-test post-test design was utilised. Previous research in this area was very limited and hence at this stage a control group was not possible due to limitations in funding from the Health Foundation and that this was primarily a service development project.

Participants were identified from adults over the age of 18 who were patients currently receiving care from either an inpatient mental health ward or the CRHTT, and presenting with suicidal ideation. Those who had reported experiencing at least one event that they considered to be traumatic and which still caused them some disturbance were offered the opportunity to participate in the project. Individuals currently experiencing an acute psychotic episode were excluded. Only individuals currently being supported by the CRHTT or an acute ward were eligible to participate in the project. Treatment as usual on the acute wards and within CRHTT mainly consisted of medication and support from a multi-disciplinary team. During participation in the study, participants were not receiving support or treatment from any other NHS service.

### Patient and public involvement

Patient were not involved in the design of the study but have subsequently been involved in the dissemination of results.

### Procedure

Patients were referred into the project from members of the multidisciplinary team on either the acute wards or CRHTT. A paper note screening was completed prior to offering an assessment and any patient not meeting the criteria for the project was excluded at this stage. Patients were assessed by the assistant psychologist with the support of the project lead within 5 days of a referral. This involved a semi structured interview to discuss the nature of the trauma and introduce the research project. Patients were assessed for their usual treatment pathway (i.e. secondary care psychological therapies, community mental health team) and the appropriate referrals were made.

EMDR therapy started within days of the initial assessment. Patients received 2–3 one and a half hour sessions per week which was generally delivered in an outpatient setting. A number of participants who were detained under the Mental Health Act on the inpatient wards and had a history of absconding were treated on the ward.

Treatment ended when the patient’s subjective units of distress reduced significantly, when they declined to continue or when progress was no longer being made. The end of treatment was usually made in collaboration between patient and therapist. There was no minimum or maximum number of sessions offered to patients. When patients moved between wards or to the community, or were discharged from acute services, treatment was not affected and continuity was maintained as long as the patient felt well enough to engage.

Psychometric measures outlined below were completed at the initial assessment (t0) after informed consent had been obtained and at the end of treatment (t1). At the final treatment session patients were re-assessed for further psychological or social needs and further referrals were made to appropriate services, with existing referrals withdrawn if required.

### EMDR protocol

The standard eight-phase protocol was used in the present research, with patients focusing on the image that represented the worst part of their traumatic experiences together with a negative cognition and associated sensations in their body. Patients focused on this material whilst focusing on external bi-lateral stimulus in the form of therapist-controlled eye movements or tactile stimulation. The type of external stimulus was dependant on patient preference.

Treatment continued until patients rated their subjective units of distress (SUDS) as zero or 1, as per EMDR standard protocol. At that point, other traumatic memories, if applicable, were worked on. If no further traumatic material was present, treatment was ended.

### Outcomes

The primary outcome measures were anxiety, depression, psychological impact of the trauma (PTSD symptoms), desire for suicide and service utilisation.

The Hospital Anxiety and Depression Scale (HADS) [46] aims to measure symptoms of anxiety and depression within the previous seven days. The HADS is a self-assessment measure consisting of 14 items, seven for the anxiety subscale (HADS-A) and seven for the depression subscale (HADS-D). Range of scores for both sub-scales is 0–21. It has been found to be reliable for detecting states of anxiety and depression, with the subscales also being a valid measure of severity of depression and anxiety. Higher scores indicate greater severity. Cronbach’s alpha for HADS-A varied from.68 to.93 (mean.83) and for HADS-D from.67 to.90 (mean.82).

The Impact of Events Scale – Revised (IES-R) [47] measures the current psychological impact of the trauma on mental state. It is a short, easily administered self-report measure of 22 questions. Although not diagnostic of PTSD it measures subjective responses to a specific trauma providing subscales measuring intrusion, avoidance and hyperarousal. Item scores are summed to give a total score ranging from 0 to 88. High levels of internal consistency have been reported (Intrusion: Cronbach’s alpha = .87–.94, Avoidance: Cronbach’s alpha = .84–.87, Hyperarousal: Cronbach’s alpha = .79–.91).

The Interpersonal Needs Questionnaire [48] (INQ) is a 15-item self-report measure that looks at the participant’s feelings of perceived burdensomeness (6 items scores ranging from 6 to 42) and thwarted belongingness (9 items scores ranging from 9 to 63). It is derived from Joiners’ Interpersonal theory of suicide which ascertains that thwarted belongingness and perceived burdensomeness are distinct but related constructs that can be measured and both are proximal causes for the desire for suicide. Cronbach’s alpha coefficients for perceived burdensomeness are .94 and for thwarted belongingness .91.

The Mental Health Confidence Scale [49] is a 16 item self-report measure and was used to determine the participant’s confidence in managing their own mental health symptoms. It is based on theories of self-efficacy and has a 3-factor scale of optimism, coping and advocacy. It is a reliable means of assessing mental health related efficacy beliefs. Scores range from 16 to 96 with Cronbach’s alpha for the total scare being .91.

Service utilisation was measured in two ways; an audit of the participant’s clinical records was conducted to objectively measure number of admissions to hospital and contacts with CRHTT. This data was compared at 12 months prior to treatment and 12 months after treatment had ended. Additionally, participant’s requirement for onward referrals for further therapy was also recorded, comparing their treatment need at initial assessment to their treatment need upon discharge from the project.

### Statistical analysis

A paired *t*-test was chosen to analyse the difference between baseline and post-intervention measures. The analyses were run as one tailed as, despite the exploratory nature of the project, it was hypothesised that the intervention would decrease the desire for suicide and reduce psychopathology. This was based on a previous case series by the lead author [45]. Additionally, a one tailed test has more statistical power than a two tailed test. The measure of effect was calculated using the following formula: d = (× 1-× 2)/mean SD.

Participants with missing scores were excluded completely from statistical analysis. Due to the large amount of missing data compared to the sample size, missing data correction would have been misleading to the final analysis of the results. Missing data was caused by some participants abruptly ending treatment after a few sessions when they felt better hence leaving treatment without completing final psychometrics. Statistical analysis was thus completed on a sample size of 57.

## Results

**Fig. 1:**
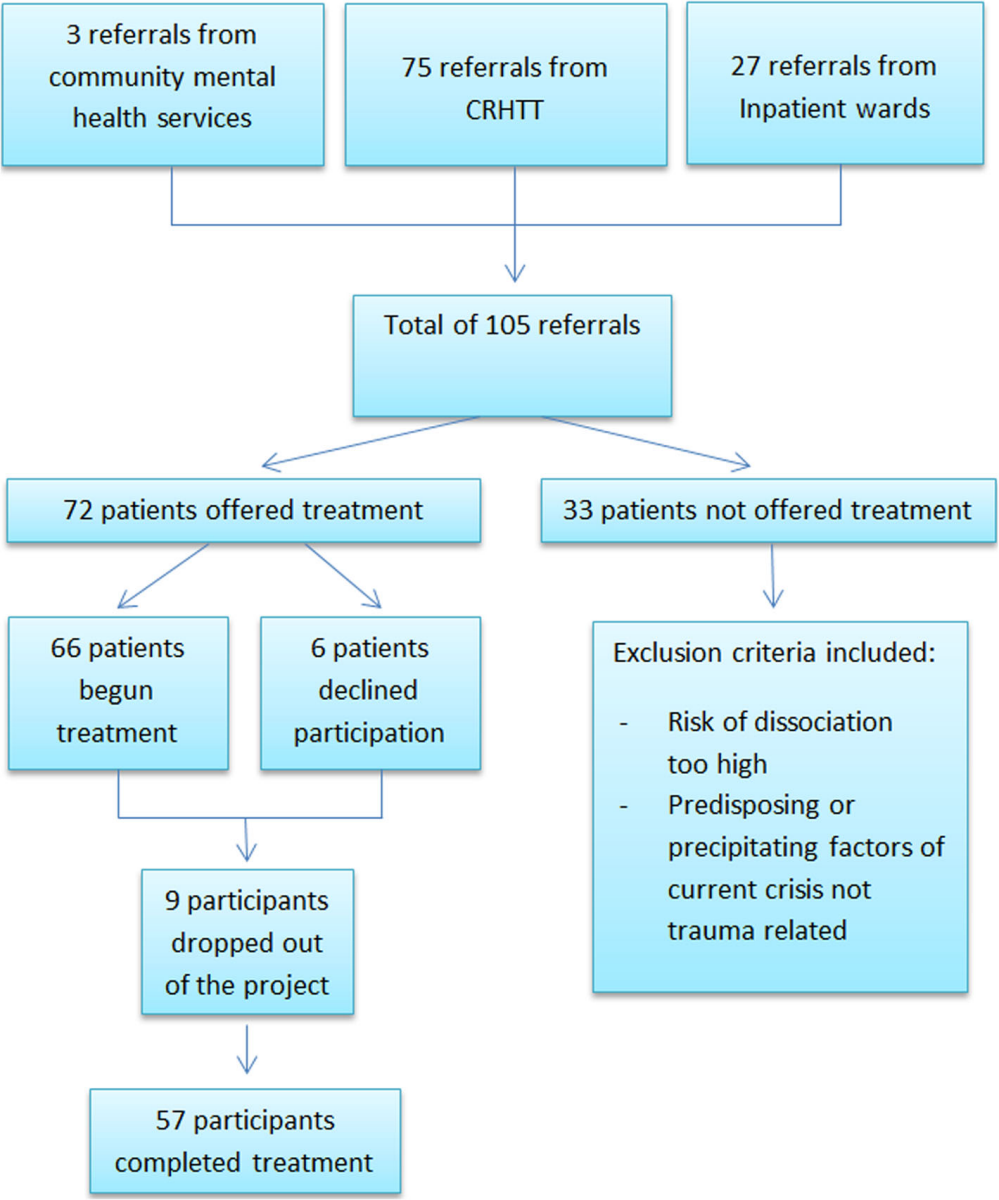
Referrals. The flow chart indicates the number and source of referrals, those included and excluded and number of participants who dropped out

Participant recruitment was continued throughout the 10-month project. Within this time a total of 105 referrals were made and following exclusion and drop out, 57 participants completed treatment (72 participants accepted for treatment) (Fig. 1).

The age range of the participants was 18–71 (mean age 34·91). There was an almost equal gender ratio of (male *n* = 29, female *n* = 28) and there was a wide range in the nature of trauma (Table 1). Participants had a variety of primary diagnoses – depression, anxiety, PTSD, complicated grief, emotionally unstable personality disorder and bipolar disorder. All had a current desire for suicide. For some this was the first time with a CRHTT or as an inpatient, but for others they had been supported in an acute care setting before.

**Table 1:** Sample demographics

	*n*
Gender	
Male	29
Female	28
Age (years)	
Range	18–71
Mean	34·91
Std Dev	12·34
Nature of trauma	
Sexual Assault	12
Childhood abuse	12
Road Traffic accident	5
Complex grief	4
Domestic Violence	3
Physical assault	3
Bullying	2
Witness severe violence, death or injury	2
Military combat trauma	2
Torture	1
Armed Robbery	1
Other	10

Participants received between 2 and 32 treatment sessions and the majority of participants required less than 12 sessions (*n* = 46, mean 8). Seven participants received between 12 and 20 session with 4 needing more than 21 sessions.

The average anxiety score post-intervention was lower than at baseline (7·84 and 16·57 respectively) (Table 2). A paired t-test showed that the difference between anxiety at baseline and post-intervention was statistically significant (t = 10·869, df = 36, *p* < ·0001, one tailed). The magnitude of the differences in the means (mean difference = 8.73, 95% CI:10·36 to 7·10) was large (d = 2·37).

**Table 2:** Results of primary outcomes

Psychometric questionnaire (construct measured)		Mean score	Standard deviation	Standard error of the mean
HADS (Anxiety)	T0	16·57	2·78	·46
	T1	7·84	4·57	·75
HADS (Depression)	T0	13·05	4·53	·75
	T1	5·27	4·03	·66
Impact of Events Scale (PTSD symptom)	T0	63·11	11·97	1·94
	T1	21·95	17·79	2·88
Interpersonal Needs Questionnaire (Perceived Burdensomeness, suicidality)	T0	43·88	13·	2·23
	T1	22·	14·45	2·48
Interpersonal Needs Questionnaire (Thwarted Belonging, suicidality)	T0	40·76	11·93	2·05
	T1	21·91	13·72	2·35
Mental Health Confidence Scale	T0	41·54	14·49	2·38
	T1	69·84	18·9	3·11

The average baseline depression score was higher than the average post-intervention score (13·05 and 5·27). The paired t-test demonstrated that the difference between depression at baseline and post-intervention was statistically significant (t = 9·111, df = 36, *p* < ·0001, one tailed). The magnitude of the differences in the means (mean difference = 7·78, 95% CI:9·52 to 6·05) was large (d = 1·82).

Average post-intervention IES-R score was lower than the average baseline IES-R score (21·95 and 63·12). A paired t-test showed that the difference between IES-R at baseline and post-intervention was statistically significant (t = 13·507, df = 37, *p* < ·0001, one tailed). The magnitude of the differences in the means (mean difference = 41·16, 95% CI:47·33 to 34·98) was large (d = 2·77).

Average INQ Perceived Burdensomeness at baseline was higher than the average score at post-intervention (43·88 and 22·00). The paired t-test demonstrated that the difference between INQ Perceived Burdensomeness at baseline and post-intervention was statistically significant (t = 7·927, df = 33, *p* < ·0001, one tailed). The magnitude of the differences in the means (mean difference = 21·88, 95% CI:26·69 to 17·07) was large (d = 1·59).

The average INQ Thwarted Belongingness baseline score was higher than the average post-intervention score (40·76 and 21·91). The paired t-test demonstrated that the difference between INQ Thwarted Belongingness at baseline and post-intervention was statistically significant (t = 9·738, df = 33, *p* < ·0001, one tailed). The magnitude of the differences in the means (mean difference = 18·85, 95% CI:23·69 to 14·01) was large (d = 1·47).

The average Mental Health Confidence score at baseline was lower than post-intervention (41·54 and 69·84). The paired t-test demonstrated that the difference between Mental Health Confidence at baseline and post-intervention was statistically significant (t = 9·738, df = 36, *p* < ·0001, one tailed). The magnitude of the differences in the means (mean difference = 28·30, 95% CI:-22·40 to − 34·19) was large (d = 1·70).

An audit of participants’ medical records showed a large reduction in both contact with CRHTT and hospital admissions in the 12 months post EMDR therapy compared to the 12 months prior (Table 3). The data shows a large reduction of over 78% for inpatient admissions and over 69% for CHRTT contacts. The number of required onward referrals also reduced for all mental health services following intervention (Table 4).

**Table 3:** Comparison of pre and post treatment service utilisation for Inpatient wards and CRHTT’s

	Inpatient admission		CRHTT Contacts	
	12 months pre treatment	12 months post treatment	12 months pre treatment	12 months post treatment
Total number	14	3	1012	306
Mean	0·28	0·06	20·24	6·12
Percentage reduction	78·57%		69·76%

**Table 4:** Comparison of initial identified treatment pathway upon assessment and the required referrals (actual referrals made) following treatment

Treatment pathway	Initial no. referrals identified	Actual referrals made
Secondary Care Psychological Therapy	29	1
Berkshire Traumatic Stress Service	9	1
Community Mental Health Team	6	1
IAPT	6	0
Total	50	3

## Discussion

Suicide is increasingly prevalent in patients under the care of mental health services and suicide prevention is high on the national agenda. Clinicians noted that patients at high risk of suicide had often experienced psychological trauma which was unresolved and led to repeated mental health crises. With little guidelines for the treatment for suicidality the researchers identified a clinical need to explore treatment options for this patient group.

The project aimed to use EMDR therapy to treat patients at high risk of suicide under the care of acute mental health services. It was hypothesised that upon completion of treatment, participants would experience less anxiety, depression and psychological distress (PTSD symptoms) as well as experience a decrease in their desire for suicide, and more able to manage their own mental health symptoms.

The above aims were achieved and our findings were in line with those hypothesised. Participants demonstrated a large reduction in anxiety, from a mean score in the severe range to a mean score in the healthy range. Similar findings were shown for participant’s feelings of depression, with a reduction from a mean score in the moderate range to a mean score in the healthy range.

Participants had a large reduction in their symptoms of PTSD and psychological distress related to the trauma. Before treatment participants exhibited symptoms at a severity indicative of a strong likelihood of PTSD. However following treatment, the mean score indicated participants’ symptoms no longer specified a diagnosis of PTSD.

Another primary focus of the research was to explore the effect of treatment on suicidality. This was achieved and participants’ feelings of perceived burdensomeness and thwarted belongingness reduced following treatment. As per Joiners model, a reduction in both of these constructs indicates a reduced desire for suicide. This reduction, coupled with a corresponding reduction in depression indicates patients were at a decreased risk of suicide after treatment with EMDR Therapy (depression being one of the main risk factors for suicide [16]).

Participants felt more able to manage their mental health symptoms and displayed higher self-efficacy following treatment. Research has indicated a link between higher scores of self-efficacy and an increased utilisation of self-help [49]. This is supported by the present findings that participants required less support from acute mental health services in the 12 months post treatment compared to the 12 months prior, and that 42% of participants exited mental health services completely following treatment.

Patients are often referred for onward services after receiving acute care and some re-present to acute mental health care whilst waiting for treatment. The project ensured that patients got treatment at their time of need, which was another contributing factor to the reduced need for onward treatment.

An unanticipated finding was that a low number of treatment sessions were required; far less than recommended by NICE guidelines. This demonstrated the feasibility of EMDR Therapy in environments such as CRHTTs and inpatient wards where length of stay is kept as low as possible. These findings, together with the large reductions in inpatient admissions and CRHTT contacts, highlight that using EMDR in this way could have a significant economic impact on health services.

There are limitations to the project design that restrict the ability to infer causation of the results. Due to the practice-based nature of the project and limited funding, no control condition was used and therefore it is unrealistic to conclude the findings are solely a result of EMDR therapy, or as a result of EMDR and treatment as usual (for example medication). Further research using a more rigorous experimental design would be recommended to identify specific contributing factors to the present results. Future research may compare EMDR therapy to treatment as usual, other evidence-based therapies such as CBT and a no treatment control. Randomisation may also be beneficial to reduce the occurrence of confounding variables which may skew the data.

The British Psychological Society [50] believe that action at an early stage must be core to any strategy for suicide prevention, and call for more large-scale clinical trials of psychological treatments to reduce suicidal ideation, suicide and suicide attempts. We echo this call. From the results presented here, immediate access to specialised treatment seems imperative in not only engaging those who are in a mental health crisis but also in effectively making a substantial difference in their recovery. Hernandez [51] reinforce what we have found both in the literature and in practical experience,

“*Despite the obvious need for an efficacious treatment for suicidal self-directed violence, the vast majority of the literature over the past 50 years has focused on the identification of risk factors and theory development*.”

## Conclusions

This research has demonstrated the feasibility of using EMDR therapy in acute mental health services. The initial findings have important clinical implications for the treatment of the desire for suicide in acute mental health services and builds upon a recent Randomised Clinical Trial using EMDR with individuals with suicidal thoughts on an inpatient ward [16]. There are a number of limitations that restrict the ability to conclude causation and therefore further research is strongly recommended. These findings introduce a trauma focused approach as an intervention in acute mental health services, and suggest EMDR therapy as a treatment option to reduce suicidality, PTSD symptoms, anxiety and depression in patients in acute mental health crises and provide significant cost savings in acute mental health care.

## Supplementary information

**Additional file 1: **Raw EMDR Data. **Table S1.** Paired Samples Statistics. **Table S2.** Paired Samples Correlations. **Table S3.** Paired Samples Test.

## Data Availability

All data generated or analysed during this study are included in this published article [and its supplementary information files].

## Abbreviations

AIP: Adaptive Information Processing
CBT: Cognitive Behavioural Therapy
CRHTT: Crisis Resolution Home Treatment Team
DBT: Dialectical Behaviour Therapy
EMDR: Eye Movement Desensitisation and Reprocessing
HADS: Hospital Anxiety and Depression Scale
IES-R: Impact of Events Scale – Revised
INQ: Interpersonal Needs Questionnaire
NHS: National Health Service
NICE: National Institute of Health and Care Excellence
PTSD: Post-Traumatic Stress Disorder
SUDS: Subjective Units of Disturbance
UK: United Kingdom
