# Supplementary material for: Using EMDR therapy with patients in an acute mental health crisis
Source: BMC Psychiatry. 2020 Jan 9;20:14. doi: 10.1186/s12888-019-2426-7 (PMC6953229; doi:10.1186/s12888-019-2426-7)
Supplement: Supplementary file 1 — Additional file 1: Raw EMDR Data. Table S1. Paired Samples Statistics. Table S2. Paired Samples Correlations. Table S3. Paired Samples Test. [file 12888_2019_2426_MOESM1_ESM.docx]

**Raw EMDR Data**

| **Table S1: Paired Samples Statistics** | | | | | |
| --- | --- | --- | --- | --- | --- |
|  | | Mean | N | Std. Deviation | Std. Error Mean |
| Pair 1 | Baseline_HADS_Anxiety | 16.5676 | 37 | 2.78429 | .45773 |
|  | Post_HADS_Anxiety | 7.8378 | 37 | 4.56748 | .75089 |
| Pair 2 | Baseline_HADS_Depression | 13.0541 | 37 | 4.53349 | .74530 |
|  | Post_HADS_Depression | 5.2703 | 37 | 4.03215 | .66288 |
| Pair 3 | Baseline_IES | 63.1053 | 38 | 11.96569 | 1.94109 |
|  | Post_IES | 21.9474 | 38 | 17.79151 | 2.88616 |
| Pair 4 | Baseline_MentalHealthConfidence | 41.5405 | 37 | 14.49060 | 2.38224 |
|  | Post_MentalHealthConfidence | 69.8378 | 37 | 18.89520 | 3.10635 |
| Pair 5 | Baseline_IPN_PercBurden | 43.8824 | 34 | 13.00295 | 2.22999 |
|  | Post_IPN_PercBurden | 22.0000 | 34 | 14.44740 | 2.47771 |
| Pair 6 | Baseline_IPN_ThwartedBelonging | 40.7647 | 34 | 11.93431 | 2.04672 |
|  | Post_IPN_ThwartedBelonging | 21.9118 | 34 | 13.72096 | 2.35312 |

| **Table S2: Paired Samples Correlations** | | | | |
| --- | --- | --- | --- | --- |
|  | | N | Correlation | Sig. |
| Pair 1 | Baseline_HADS_Anxiety & Post_HADS_Anxiety | 37 | .187 | .269 |
| Pair 2 | Baseline_HADS_Depression & Post_HADS_Depression | 37 | .268 | .109 |
| Pair 3 | Baseline_IES & Post_IES | 38 | .251 | .128 |
| Pair 4 | Baseline_MentalHealthConfidence & Post_MentalHealthConfidence | 37 | .465 | .004 |
| Pair 5 | Baseline_IPN_PercBurden & Post_IPN_PercBurden | 34 | .500 | .003 |
| Pair 6 | Baseline_IPN_ThwartedBelonging & Post_IPN_ThwartedBelonging | 34 | .423 | .013 |

| **Table S3: Paired Samples Test** | | | | | | | | | |
| --- | --- | --- | --- | --- | --- | --- | --- | --- | --- |
|  | | Paired Differences | | | | | t | df | Sig. (2-tailed) |
|  |  | Mean | Std. Deviation | Std. Error Mean | 95% Confidence Interval of the Difference | |  |  |  |
|  |  |  |  |  | Lower | Upper |  |  |  |
| Pair 1 | Baseline_HADS_Anxiety - Post_HADS_Anxiety | 8.72973 | 4.88563 | .80319 | 7.10078 | 10.35868 | 10.869 | 36 | .000 |
| Pair 2 | Baseline_HADS_Depression - Post_HADS_Depression | 7.78378 | 5.19687 | .85436 | 6.05106 | 9.51651 | 9.111 | 36 | .000 |
| Pair 3 | Baseline_IES - Post_IES | 41.15789 | 18.78402 | 3.04717 | 34.98374 | 47.33205 | 13.507 | 37 | .000 |
| Pair 4 | Baseline_MentalHealthConfidence - Post_MentalHealthConfidence | -28.29730 | 17.67589 | 2.90590 | -34.19073 | -22.40386 | -9.738 | 36 | .000 |
| Pair 5 | Baseline_IPN_PercBurden - Post_IPN_PercBurden | 21.88235 | 13.78573 | 2.36423 | 17.07228 | 26.69242 | 9.256 | 33 | .000 |
| Pair 6 | Baseline_IPN_ThwartedBelonging - Post_IPN_ThwartedBelonging | 18.85294 | 13.86763 | 2.37828 | 14.01430 | 23.69158 | 7.927 | 33 | .000 |
